# Discontinuation of thyroid hormone treatment among children in the United States with congenital hypothyroidism: findings from health insurance claims data

**DOI:** 10.1186/1471-2431-10-9

**Published:** 2010-02-15

**Authors:** Alex R Kemper, Lijing Ouyang, Scott D Grosse

**Affiliations:** 1 Program on Pediatric Health Services Research, Department of Pediatrics, Duke University Medical Center, Durham, NC, USA; 2 National Center on Birth Defects and Developmental Disabilities, Centers for Disease Control and Prevention, Atlanta, GA, USA

## Abstract

**Background:**

Thyroid hormone treatment in children with congenital hypothyroidism can prevent intellectual disability. Guidelines recommend that children diagnosed with congenital hypothyroidism through newborn screening remain on treatment to at least 3 years of age, after which a trial off therapy can determine which children have transient hypothyroidism. The purpose of this study was to describe the rate at which children with congenital hypothyroidism in the United States discontinue thyroid hormone treatment in early childhood.

**Methods:**

Retrospective analysis of the 2002-2006 MarketScan^® ^Commercial Claims and Encounters research databases and the 2001-2005 MarketScan Multi-State Medicaid databases. Children were classified as having congenital hypothyroidism based on billing codes and having filled a prescription for thyroid hormone treatment. Kaplan-Meier curve analysis was used to determine discontinuation rates.

**Results:**

There were a total of 412 Medicaid-enrolled children and 292 privately-insured children with presumed congenital hypothyroidism included in this study. The overall birth prevalence of congenital hypothyroidism across both datasets was about 1 per 2,300. By 36 months, the percentage who had discontinued thyroid replacement treatment was 38% (95% Confidence Interval: 32%-44%). Medicaid-enrolled children had a more rapid decline in the first 24 months of treatment compared to those with private insurance (*P *= 0.02).

**Conclusions:**

More than one-third of children treated for congenital hypothyroidism discontinued treatment within 36 months, which is inconsistent with current guidelines. It is not known how many of these children required continued treatment or experience adverse effects from discontinuation. These findings emphasize the critical need for follow-up systems to monitor the outcome of newborn screening.

## Background

Although there has been dramatic expansion in the conditions identified by newborn screening, few data are available regarding the care that children receive after they are diagnosed through newborn screening. Appropriate delivery of health care services is necessary to assure the benefit of early diagnosis. We chose to focus on congenital hypothyroidism (CH) because of its relatively high prevalence compared to other conditions included in newborn screening and because treatment is among the most straight-forward.

Newborn screening for CH followed by the early initiation of thyroid hormone treatment to normalize thyroxin (T_4_) concentrations has been shown to prevent intellectual disability (i.e., mental retardation) [1-3]. Among population-based studies of children born prior to the introduction of newborn screening for CH in the late 1970s, 8% to 29% of children with clinically diagnosed CH received special education services for intellectual disability [4-6].

Despite the unquestioned public health success from newborn screening and the management of CH, there are a number of gaps in knowledge. For example, there has been a sustained rise in the reported frequency of CH among infants in the United States (US) from 24.4 per 100,000 in 1987 to 42.2 per 100,000 in 2002[7]. It is unknown whether this reflects a real increase[8]. In Australia, increased birth prevalence of CH has been tied to changes in diagnostic criteria and clinical practice[9].

One important challenge in understanding the epidemiology of CH is that some newborns will have transient CH, a temporary depression of thyroid hormone concentrations that can last from several days to several months[8]. If confirmatory testing during the first month indicates CH, US guidelines call for children to remain on treatment to at least 3 years of age, after which a trial of withdrawal off treatment can determine which children have transient hypothyroidism[10]. Studies from other countries have reported that 10 to 15% of children treated for CH ultimately prove to not need treatment past 3 years of age to maintain normal hormone concentrations, and thus have transient hypothyroidism[11,12]. This percentage could be higher in the US, particularly if physicians are more likely to assign a diagnosis and prescribe thyroid hormone therapy, which is considered safe and inexpensive, while waiting for more definitive test results. Little is known about how many children in the US have either permanent or transient CH.

It is not known how many children treated for CH discontinue treatment, with or without physician supervision. The purpose of this study was to explore the birth prevalence of presumed CH, the relationship of presumed CH to sex, and the rate at which children diagnosed with CH in the US discontinue thyroid hormone treatment in early childhood. Children who discontinued treatment may have had either transient CH or a mild form of permanent CH.

## Methods

The study design was a retrospective analysis of health insurance claims databases in which infants treated for CH were followed over time to determine when treatment stopped based on the lack of thyroid hormone treatment prescription refills. We used health insurance claims data to conduct a retrospective cohort study of infants with presumed CH to track use of thyroid hormone treatment by individuals with continued enrollment in the same health plan.

### Data Sources

We analyzed two different sources of data, the MarketScan Commercial Claims and Encounters research databases for the years 2002-2006 and the MarketScan Multi-State Medicaid databases for the years 2001-2005 from Thomson Reuters. The MarketScan research databases contain information on inpatient, outpatient, and prescription drug claims for enrolled individuals which allow for tracking of services received by the same individuals over time as long as they remain enrolled. Two of us (S.D.G., L.O.) initially analyzed data on privately-insured children from the Commercial database consisting and presented preliminary findings at a Centers for Disease Control and Prevention workshop in February 2008[13]. The present study replicated that analysis with an analysis of the independently collected MarketScan Multi-State Medicaid data.

The MarketScan Commercial databases contain information from the private health insurance plans of about 100 self-insured employers each year, including large private employers and state governments. We excluded those plans that do not submit pharmacy claims. Drug claims in the Commercial database include claims where individual copays fully met the pharmacy cost with no additional health plan reimbursement. The Multi-State Medicaid databases contain claims data from eight unidentified states and include drug claims data for all enrollees. Unlike the Commercial database, few Medicaid drug claims were associated with copays.

Although drug claims are well-coded, specific laboratory tests (e.g., thyroid stimulating hormone [TSH], free T_4_) or imaging studies (e.g., thyroid ultrasound, thyroid nuclear medicine scans) do not always appear properly coded within the databases. To protect subject confidentiality, both the Multi-State Medicaid and Commercial databases include only birth year but not exact birth date. In our experience, race and ethnicity are not reliably coded in these databases[14]. The only other demographic variable is sex.

### Birth Prevalence of Presumed Congenital Hypothyroidism

We considered a child to have presumed CH if they had at least one outpatient claim for CH (*International Classification of Diseases, Ninth Revision *[ICD-9] code 243) and one or more filled prescriptions for thyroid hormone therapy during their first year of life. The overall birth prevalence was calculated separately for Medicaid-enrolled children and privately insured children as the percentage of newly diagnosed and treated cases of CH among all children born during the study years. We calculated the 95% confidence interval (CI) based on the expected binomial distribution of this count data.

### Rate of Thyroid Hormone Treatment Continuation

Kaplan-Meier analysis was used to evaluate the rate of continuation of thyroid hormone treatment by number of months since the initiation of treatment adjusted for censoring due to individuals discontinuing health plan enrollment or coverage. This was based on the total number of months for which treatment was received, as assessed by cumulative duration of prescription being filled, by children who were born in 2001-2005 in the Multi-State Medicaid databases or who were born in 2002-2006 in the Commercial databases. We were not able to determine the ages in months over which treatment was received because of the lack of exact birth dates in the databases.

Throughout our analyses, we made a conservative assumption that would bias towards longer rates of treatment. The quantity of drug supplied is not recorded in pharmacy claims. Because prescriptions can be filled for up to 3 months and families may have some extra supply at home due to skipped doses, children may continue on treatment for several months after the last claim is filed. Therefore, we considered thyroid hormone treatment to be discontinued if there were no prescription claims by the fourth month after the last prescription was filled. In the Kaplan-Meier analysis, individuals were right censored if they were no longer in the claims databases during the fourth month after the last prescription refill or if the fourth month fell outside of the study period. We constructed Kaplan-Meier survival curves to estimate the probability of continued thyroid hormone treatment at each month since initiation of treatment stratified by insurance type (Medicaid vs. private health insurance). To evaluate the replication of our analyses in the two independent datasets, we used the log-rank test for equality of Kaplan-Meier functions.

We similarly evaluated the relationship between sex and discontinuation. We hypothesized that females were more likely to continue thyroid hormone treatment because the prevalence of permanent congenital hypothyroidism is about twice as high among females than males[12]. In contrast, transient or very mild permanent CH is equally common among females and males[12].

### Data Analysis

All analyses were conducted with Stata 10 statistical software (College Station, TX). We considered *P *< 0.05 to be statistically significant. This study was approved by the Duke University Medical Center institutional review board. No other approval was required for the use of the claims data.

## Results

### Prevalence of Presumed Congenital Hypothyroidism

There were a total of 412 Medicaid-enrolled children born during the years 2001-2005 who met the case definition for presumed CH, yielding a birth prevalence of 47 per 100,000 (95% CI: 42 - 51 per 100,000). A total of 292 privately-insured children born during the years 2002-2006 met the case definition for presumed CH, for a similar birth prevalence (44 per 100,000 [95% CI: 39-49 per 100,000]). The birth prevalence was approximately 1 in 2,300 in both cohorts.

### Rate of Thyroid Hormone Continuation

**Figure 1:**
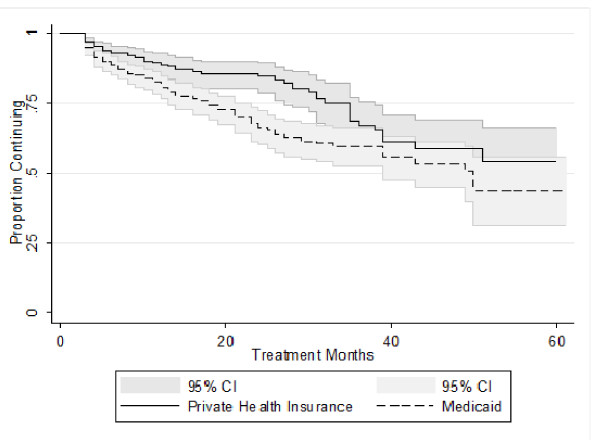
**Kaplan-Meier Curve for the Continuation of Thyroid Hormone Treatment for Children with Presumed Congenital Hypothyroidism, by Insurance Type**.

Among the Medicaid-enrolled children with presumed CH, there were a total of 7,023 person-months of thyroid hormone treatment compared to 5,202 person-months of thyroid hormone treatment among those with private health insurance. Figure 1 presents a Kaplan-Meier curve for the continuation of thyroid hormone treatment among the Medicaid-enrolled and privately insured cases of presumed CH. Table 1 presents the cumulative rate of discontinuation in six-month intervals through 42 months of treatment. Although Medicaid-enrolled children had a more rapid initial decline in the rate of continuation in the first 24 months of treatment compared to those with private insurance (*P *= 0.02), by 36 months the percentage who had discontinued thyroid hormone treatment was similar between Medicaid-enrolled (40% [95% CI: 34%-48%]) and privately insured children (33% (95% CI: 24%-44%). The overall percentage who had discontinued by 36 months was 38% (95% CI: 32% - 44%).

**Table 1:** Rates of Cumulative Thyroid Hormone Discontinuation for Children with Presumed Congenital Hypothyroidism by Insurance Type in Six-Month Increments from Initiation of Therapy

Duration	Medicaid Rate (95% CI*)	Private Health Insurance Rate (95% CI*)
6 Months	11% (9%-15%)	7% (5%-11%)
12 Months	19% (15%-24%)	11% (8%-16%)
18 Months	26% (21%-31%)	14% (10%-20%)
24 Months	34% (28%-40%)	15% (10%-21%)
30 Months	38% (32%-45%)	21% (15%-28%)
36 Months	40% (34%-48%)	33% (24%-44%)
42 Months	45% (37%-52%)	39% (29%-50%)

*CI: Confidence Interval

### Relationship of Presumed CH to Sex

In the birth year, females represented 53% (n = 368) of all cases (i.e., ratio of females to males of 1.1:1). Males were more likely to discontinue thyroid hormone treatment (*P *= 0.04) over time. Among those that continued for more than 36 months, the ratio of females to males was 1.8:1 (95% CI: 1.14:1 - 2.98:1).

## Discussion

To our knowledge, this is the first study to examine rates of discontinuation of thyroid hormone therapy among a broad cross-section of US children diagnosed with CH. In contrast, the Texas newborn screening follow-up program found that very few children with CH managed by pediatric endocrinologists are reclassified as having transient CH[8]. The finding that approximately 40% of children with CH appear to discontinue treatment by 36 months after initiation of treatment was unexpected. We are reassured that these results are correct based on agreement with the accepted birth prevalence of CH and the concordance of our replicated findings. We found a birth prevalence of presumed CH of about 1 in 2,300 in both samples, consistent with the prevalence of CH reported from U.S. newborn screening records[7,15]. We also found similar rates of thyroid hormone treatment discontinuation independently in both samples. However, the patterns differed significantly by duration of treatment, with higher period-specific discontinuation rates before 24 months in the Medicaid sample and higher period-specific discontinuation rates in the Commercial sample after 24 months. We do not know the underlying causes of this difference.

According to current guidelines, children with confirmed CH should remain on treatment until at least 36 months of age. Only then should those who are not known to have an absent or dysplastic thyroid gland undergo carefully monitored trial withdrawal of therapy[10]. We suspect that discontinuation of thyroid hormone treatment within six months of its start represents cases where presumptive treatment was begun based on preliminary test results and then stopped after confirmatory testing ruled out CH. The 5 to 15% discontinuation rate during the first 6 months is consistent with that interpretation. However, between 6 and 36 months few children should undergo discontinuation if parents and physicians follow the CH management guidelines. Our findings of discontinuation during this period appear inconsistent with the guidelines. It may be that families are stopping treatment and that their children do not receive monitoring to assure that thyroid hormone concentrations are in the normal range. This hypothesis is consistent with the preliminary findings of a survey of families of 23 children with borderline CH conducted by the Michigan Department of Community Health newborn screening follow-up program, seven of whom (30%) reported stopping treatment on their own, without medical advice[16].

We are concerned that an important number of children for whom treatment was discontinued likely had permanent CH, and that these children should have remained on treatment. If so, it is likely that such children may have milder forms of CH, for which the effects of thyroid hormone treatment discontinuation may not be readily apparent.

As expected, we found that children treated for CH who stopped treatment within roughly 3 years of beginning treatment have a nearly balanced sex ratio. In contrast, those who continued treatment past 36 months had a roughly two to one ratio of females to males, as is characteristic of children with permanent CH in general [9,17-19] and those with thyroid dysgenesis in particular[12,20]. These findings suggest that those who discontinued early had false positive newborn screening results or transient hypothyroidism, and that the bulk of those who continued treatment had permanent CH. However, these data do not provide insight into how many children may have appropriately or inappropriately stopped receiving thyroid hormone treatment.

### Limitations

Our study had several limitations. One of these is common to both research databases. First, for most children we were not able to ascertain which laboratory tests were conducted and we had no information on the results of laboratory tests. Two other limitations were specific to the MarketScan Commercial database sample: high rates of attrition due to disenrollment and the possibility that co-pays for prescriptions might have led to prescriptions being filled elsewhere, perhaps reimbursed by a different parent's health plan. Neither of these limitations applies to the MarketScan Multi-State Medicaid database. The similarity in findings between the analyses of data from two independent samples strengthens the credibility of the findings.

## Conclusions

Long-term follow-up of US children diagnosed with CH and other disorders detected by newborn screening is needed to assure the continued receipt of appropriate treatment and the full realization of the health gains from early detection of inborn errors. Although approximately 80% of cases of CH are sporadic rather than genetic in origin,[21] close management and monitoring of genetic disorders such as sickle cell disease is also essential. Limited adherence to penicillin prophylaxis among children with sickle cell disease is widely reported [22-24]. As a result, the potential to prevent invasive pneumococcal disease and associated mortality through newborn screening was realized only in part. The subsequent introduction in 2000 of the heptavalent pneumococcal conjugate vaccine was associated with a greater than 40% reduction in sickle cell disease-related mortality among Black or African-American children under 5 years of age[25].

It appears that many US children with CH may have treatment discontinued inappropriately, although this does not appear to be the case among children with CH managed by endocrinologists[8]. This could potentially cause long-term harm for at least some children with permanent CH. Unfortunately, newborn screening program records are inadequate for the surveillance of CH, both to assess prevalence and time trends. Without additional data, we cannot determine whether the reported increase in CH prevalence in the US in recent decades [7] is real or artifactual, or the degree to which discontinuation of thyroid hormone treatment may be appropriate. Public health responsibility requires the collection of long-term follow-up data [26] to address these fundamental questions. Such data need to include findings from laboratory tests of thyroid hormone status and be representative of all children with CH, not just those managed by pediatric endocrinologists. Developing a system to evaluate health services delivery and health outcomes for individuals diagnosed with CH through newborn screening would be an important model for monitoring the impact of newborn screening more broadly. In the meantime, practical interventions are required to assure adherence to available guidelines. To help with this, the US Health Resources and Services Administration has developed a national network of regional coordinating centers to disseminate guidelines and work with primary care providers, specialists, and families to improve care delivery for children who had a positive newborn screen[27].

## Abbreviations

(CI): confidence interval; (CH): congenital hypothyroidism; (ICD-9): *International Classification of Diseases, Ninth Revision*; (TSH): thyroid stimulating hormone; (T_4_): thyroxin; (US): United States

## Competing interests

The authors declare that they have no competing interests.

## Authors' contributions

ARK participated in the study design, conducted analyses, contributed to the discussion, and wrote the manuscript with SDG. LO participated in data analysis, contributed to the discussion, and reviewed/edited the manuscript. SDG designed the study, obtained the data, participated in data analysis, contributed to the discussion, and wrote the manuscript with ARK. All authors read and approved the final manuscript.

## Pre-publication history

The pre-publication history for this paper can be accessed here:

http://www.biomedcentral.com/1471-2431/10/9/prepub
